# Epidemiological and time series analysis on the incidence and death of AIDS and HIV in China

**DOI:** 10.1186/s12889-020-09977-8

**Published:** 2020-12-14

**Authors:** Bin Xu, Jiayuan Li, Mengqiao Wang

**Affiliations:** Department of Epidemiology and Biostatistics, West China School of Public Health and West China Fourth Hospital, Sichuan University, Renmin South Road 16, Chengdu, Sichuan Province 610041 PR China

**Keywords:** AIDS, HIV, Incidence, Death, ARIMA model

## Abstract

**Background:**

To investigate the regional and age-specific distribution of AIDS/HIV in China from 2004 to 2017 and to conduct time series analysis of the epidemiological trends.

**Method:**

Using official surveillance data from publicly accessible database of the national infectious disease reporting system, we described long-term patterns of incidence and death in AIDS/HIV, analyzed age group and regional epidemic characteristics, and established Autoregressive Integrated Moving Average (ARIMA) models for time series analysis.

**Result:**

The incidence and death of AIDS/HIV have increased rapidly from 2004 to 2017, with significant difference regarding age groups and provincial regions (a few provinces appear as hot spots). With goodness-of-fit criteria and using data from 2004 to 2015, ARIMA (0,1,3) × (2,0,0), ARIMA (3,1,0) × (1,0,1), and ARIMA (0,1,2) × (2,0,0) were chosen as the optimal model for the incidence of AIDS, HIV, and combined; ARIMA (0,1,3) × (1,0,0) was chosen as the optimal model for the death of AIDS, HIV, and combined. ARIMA models robustly predicted the incidence and death of AIDS/HIV in 2016 and 2017.

**Conclusion:**

A focused intervention strategy targeting specific regions and age groups is essential for the prevention and control of AIDS/HIV. ARIMA models function as data-driven and evidence-based methods to forecast the trends of infectious diseases and formulate public health policies.

**Supplementary Information:**

The online version contains supplementary material available at 10.1186/s12889-020-09977-8.

## Background

Acquired immunodeficiency syndrome (AIDS) is a serious infectious disease caused by the human immunodeficiency virus (HIV), which mainly invades the immune system (particularly on CD4+ T lymphocytes, monocyte macrophages, and dendritic cells) [1]. The main manifestation of AIDS is the gradual to complete deficiency of immune response to the various opportunistic infections, tumors, and abnormities [2]. When healthy individuals initially get infected with HIV, they become HIV carriers, but it may generally take years for most carriers to develop AIDS symptoms when they become AIDS patients. Nevertheless, both HIV carriers and AIDS patients are capable of infecting others with HIV through body fluids (blood, semen fluid, vaginal fluid, breast milk etc.) [3]. With few therapeutical drugs or treatments available, AIDS/HIV remains one of the leading causes of morbidity and mortality worldwide, and poses huge challenges for public health and population wellness both in developing and developed countries [4, 5]. By 2018, there has been approximately 76.1 million people living with HIV, and 35 million people have already died of AIDS-related diseases [6].

China faces tough challenges in controlling AIDS/HIV: all 31 provincial regions in mainland China had recorded HIV cases by 1998 [7], and there are 64,170 new AIDS patients and 18,780 AIDS-related deaths in 2018 alone [8]. New incidences demonstrate the transmission from high-risk groups to the general population, and an increasing number of HIV carriers begin to develop clinical AIDS [9]. National government implemented the “Four Frees and One Care” policy (free treatment, free voluntary counseling and testing, free prevention of mother-to-child transmission, free schooling for AIDS orphans, and provision of social relief for AIDS/HIV patients) in 2004, and has been continuously tackling this public health challenge. However, despite years of efforts and accomplishments, HIV infection has been rising in China. The Joint United Nations Programme on AIDS/HIV (UNAIDS) proposes an ambitious “90–90-90” plan: by 2020, 90% of the HIV-infected would be diagnosed, 90% of HIV carriers would receive antiviral therapy, and 90% of the patients under treatment would have viral suppression [10]. Epidemiology speaks a hard if not impossible reality for most countries worldwide (including China) to achieve such high-bar goals.

To better understand the epidemiological trends and patterns of infectious diseases, it is essential to monitor and analyze the incidence and death on a long-term perspective. Mathematical models such as SEI (susceptible-exposed-infected) were applied to study the AIDS/HIV epidemic in China [11, 12], and other studies have reported on the epidemic characteristics [13, 14]. Nevertheless, there lacks systematical efforts to model the incidence and death of AIDS/HIV, to measure the performance of the predictive models, and to evaluate the data-driven approach in informing policy makers and influencing resource allocation. Time series analysis builds upon historical data to both extract the underlying time-dependent structure and forecast future developments. Autoregressive Integrated Moving Average (ARIMA) models overcome the limitation of regression analysis and investigate seasonal fluctuation of linear trend and random error [15]. ARIMA has been successfully applied to the analysis of various infectious diseases including influenza [16], malaria [17], hand-foot-and-mouth [18], tuberculosis [19], and hepatitis [20].

China CDC (Centers for Disease Control and Prevention) records and releases annual/monthly incidence and death data of AIDS and HIV, offering the data platform for a detailed perspective on the epidemiology of AIDS/HIV in China. This study aims to retrospectively analyze the annual incidence and death of AIDS/HIV in China from 2004 to 2017, at both national and regional levels, and for stratified analysis on age subgroups. It further fit the ARIMA models on the monthly incidence and death trends to formulate the long-term infection pattern and forecast developing trends in the near future. Such evidence-based information and knowledge would benefit the institution and implementation of urgent public health actions for the effective prevention and control of the AIDS/HIV in the most populous country on earth. Additionally, this study demonstrates the essence of learning from historical data, fitting the known trends to time series models, and predict in advance for future patterns and hot spots. Data science should and will be a key component of the epidemiological research that aims to tackle the most challenging infectious diseases confronting human populations.

## Methods

### Data source

This study used officially released and publicly available data on the incidence and death statistics of infectious diseases from the Data Center of China Public Health Science database (http://www.phsciencedata.cn/Share/en/) hosted by the Chinese Center for Disease Control and Prevention (China CDC). Annual/monthly incidence and death data of AIDS and HIV (AIDS for HIV-positive AIDS patients, and HIV for HIV-carriers without AIDS symptoms) from 2004 to 2017 were recorded and released by the National Center for AIDS/STD Control and Prevention (NCAIDS/STD) of China CDC. Accompanied data on the epidemiological characteristics (geographical and age-specific) were also used. Incidence and death of undetermined provincial regions or ages were excluded in the analysis, but such cases were rare (< 0.1% of total counts).

### Data availability and reproducible research

All data used in this study were publicly available from the web-based database as described in the *Data source* section. R code of ARIMA analysis was publicly accessible through the GitHub page of the corresponding author Mengqiao Wang (https://github.com/westchinabiomedicaldatascience-Wang-lab/AIDS-HIV_ARIMA).

### Data analysis

All data analysis was conducted in the R statistical environment (version 4.0.2, R Core Team). A collection of extended packages (*forecast*, *tidyverse*, *sp*, *maptools* etc.) were used throughout the project. A significance level of 0.05 was used for null-hypothesis tests.

### ARIMA model

Autoregressive Integrated Moving Average (ARIMA) is an analytical method that formulates time series values as linear functions of past values and random errors [21]. ARIMA model facilitates the decomposition of time series into general trends, cyclical patterns, and random fluctuations. For a model of ARIMA(p, d, q) × (P, D, Q)_S_, (p, d, q) and (P, D, Q) are the respective orders of non-seasonal and seasonal components, and s represents the seasonal period (in this study, s = 12 for 12 months). ARIMA modeling comprises of stationary model fitting, parameter estimation, and model diagnosis [22]. Stationary sequence is the precondition of ARIMA analysis, and could be validated by the augmented Dickey-Fuller test (ADF) [23]. For non-stationary sequence, the calculation of difference followed by the Box-Cox transformation could stabilize the mean and the variance, for which the degree of difference is the order d in the ARIMA(p, d, q) model. The full dataset of incidence and death of AIDS, HIV, and combined is divided into two parts: data from 2004 to 2015 were used as historic information to train and fit ARIMA models, while data in 2016 and 2017 were withheld as “pseudo-unknown” values to forecast such information by the chosen ARIMA and as well evaluate model performance. Selection of seasonal and nonseasonal orders were facilitated with reference to the auto-correlation and partial auto-correlation function plots [24]. A combinatorial spectrum of parameters were compared in parallel for ARIMA models using *forecast::auto.arima()* function in R environment, and an optimal set of parameters was chosen by the minimum Akaike’s information criterion (AIC) and Bayesian information criterion (BIC).

Coefficients in ARIMA models are calculated using the method of maximum likelihood estimation (MLE). In model fitting, the residual sequence was checked to qualify as white noise sequence by the Ljung–Box test. Diagnostic checking parameters including the mean absolute scaled error (MASE), root mean square error (RMSE), Akaike Information Criterion (AIC), and Bayesian Information Criterion (BIC) were used to evaluate the goodness-of-fit of constructed models [25, 26].

## Results

### Epidemiological trends of AIDS/HIV in China

**Fig. 1:**
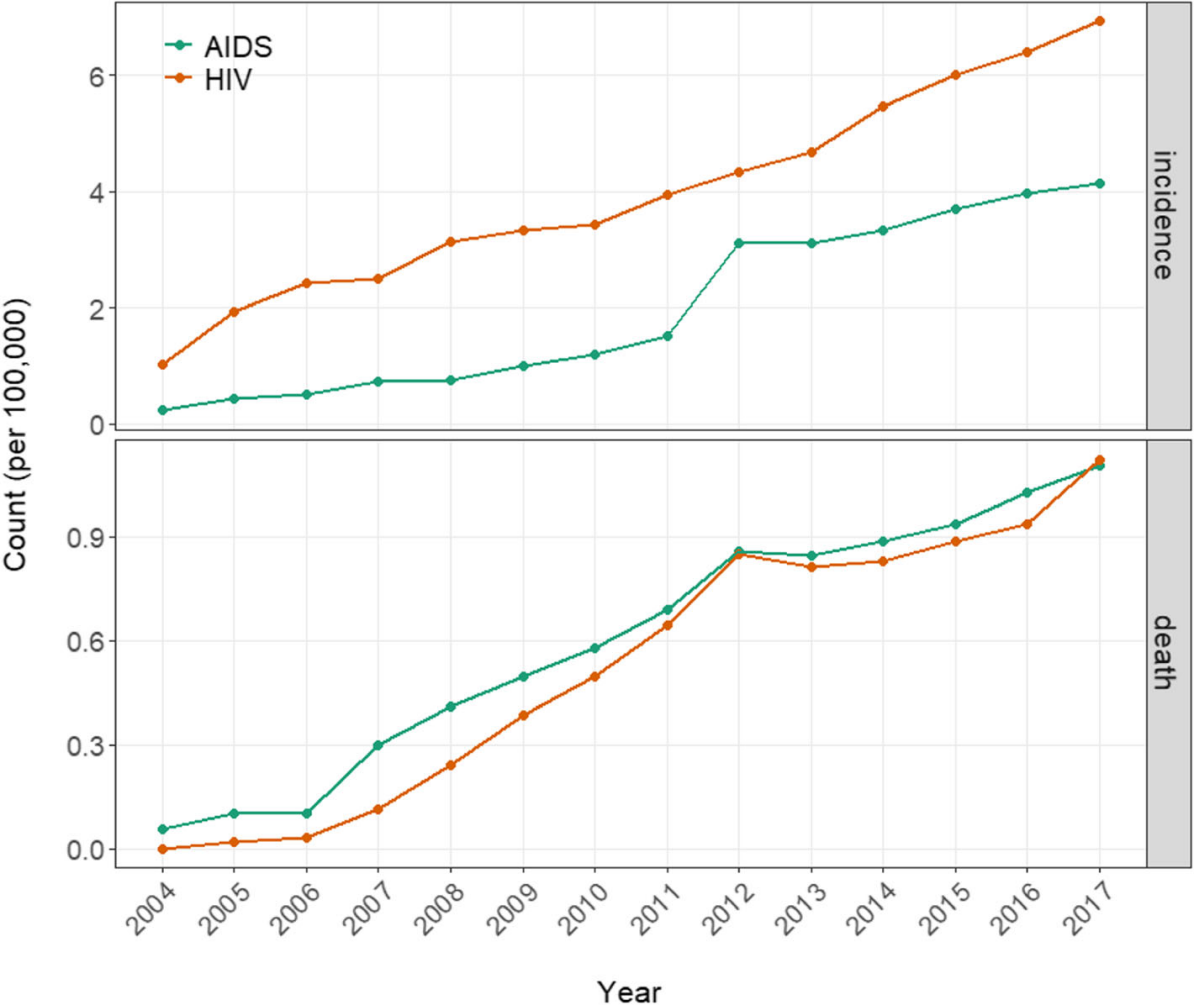
Incidence and death counts (per 100,000) of AIDS/HIV in China from 2004 to 2017

In the recent time range from 2004 to 2017, China underwent a steadily deteriorating public health challenge of AIDS/HIV at the national level (Table 1 and Figure S1). In this 14-year period, annual total incidence of AIDS patients and HIV carriers increased more than 18 and 7 folds respectively; the jumps in annual total deaths were even more striking. By adjusting the slightly growing population, such trends were still clearly present for the incidence and death for AIDS/HIV as per 100,000 individuals (Fig. 1). Unlike other infectious diseases such as influenza and hand-foot-and-mouth disease, monthly incidence and death of AIDS/HIV were largely non-cyclical and displayed no typical seasonal patterns (Figure S2). However, an ascent in November/December followed by a drop in January/February was consistently observed.

**Table 1:** Statistics of AIDS/HIV from 2004 to 2017 in China

Year	AIDS		HIV	
	Incidences	Deaths	Incidences	Deaths
2004	3054	741	13,258	2
2005	5621	1316	25,266	281
2006	6671	1331	31,591	392
2007	9727	3904	32,906	1515
2008	10,059	5389	41,466	3214
2009	13,281	6596	44,192	5142
2010	15,982	7743	45,640	6605
2011	20,450	9224	52,746	8620
2012	41,929	11,575	58,399	11,467
2013	42,286	11,437	63,498	10,974
2014	45,145	12,030	74,048	11,224
2015	50,330	12,755	81,696	12,072
2016	54,360	14,091	87,764	12,833
2017	57,194	15,251	95,549	15,467

### Epidemiological trends of AIDS/HIV in provincial regions

**Fig. 2:**
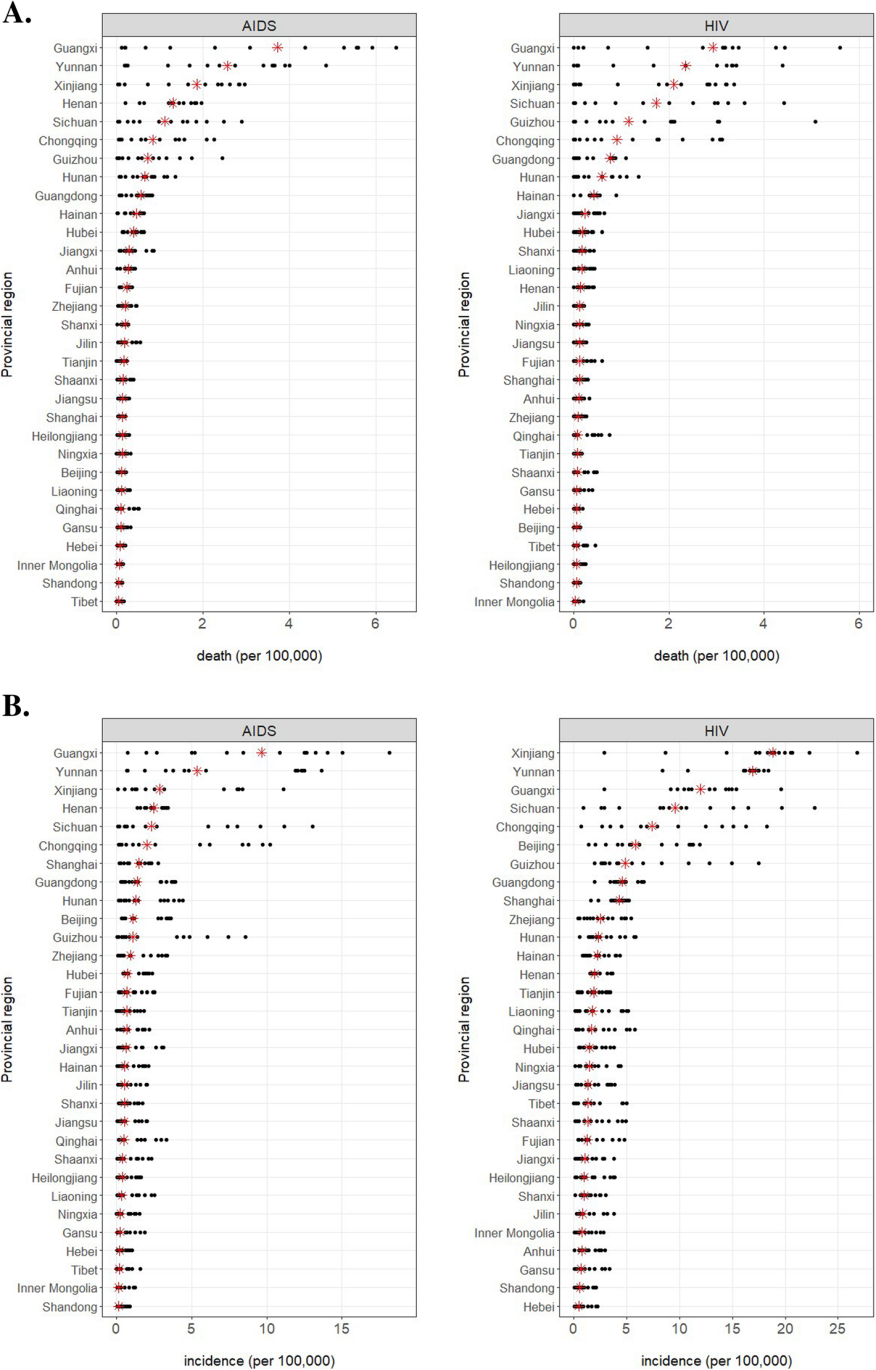
Incidence (**a**) and death (**b**) (per 100,000) of AIDS/HIV in 31 provincial regions from 2004 to 2017. The red asterisks annotate the region-wise 14-year median

**Fig. 3:**
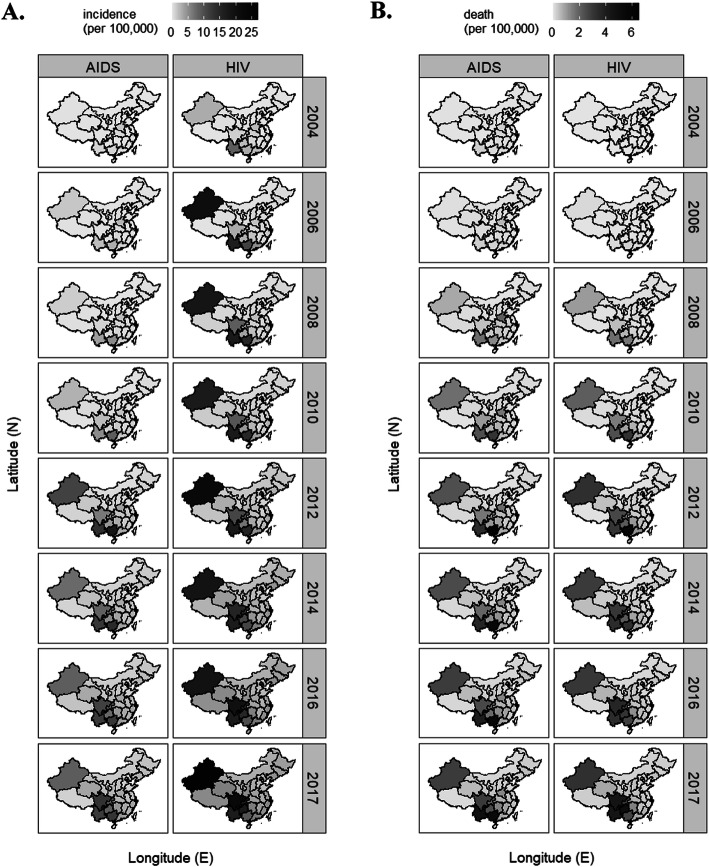
Incidence (**a**) and death (**b**) (per 100,000) of AIDS/HIV in 31 provincial regions from 2004 to 2017 as gradient-scaled maps of China. Note: to save space, only even years and the most recent year of 2017 were displayed. Odd years omitted were displayed in Fig. S3. Shaded provincial regions were comparable in Fig. 3 and Fig. S3 as the same color scale was applied in these two figures

Zooming from the national to the provincial level, geographical epidemiology reveals a significant discrepancy in the regional incidence (Fig. 2a) and death (Fig. 2b) of AIDS/HIV. The hotbed provincial regions were geographically clustered into 3 segments: 1). the southwestern region including Guangxi, Yunnan, Guizhou, Sichuan, and Chongqing, 2). the northwestern region of Xinjiang, and 3). a central province of Henan (Figs. 3S3 and S4). The disproportional distribution of AIDS/HIV incidence and death reveals the list of “epicenter” provincial regions and underlies the importance in diversified policy administration and resource allocation for the prevention and control of AIDS/HIV in China.

### Epidemiological trends of AIDS/HIV in age groups

**Fig. 4:**
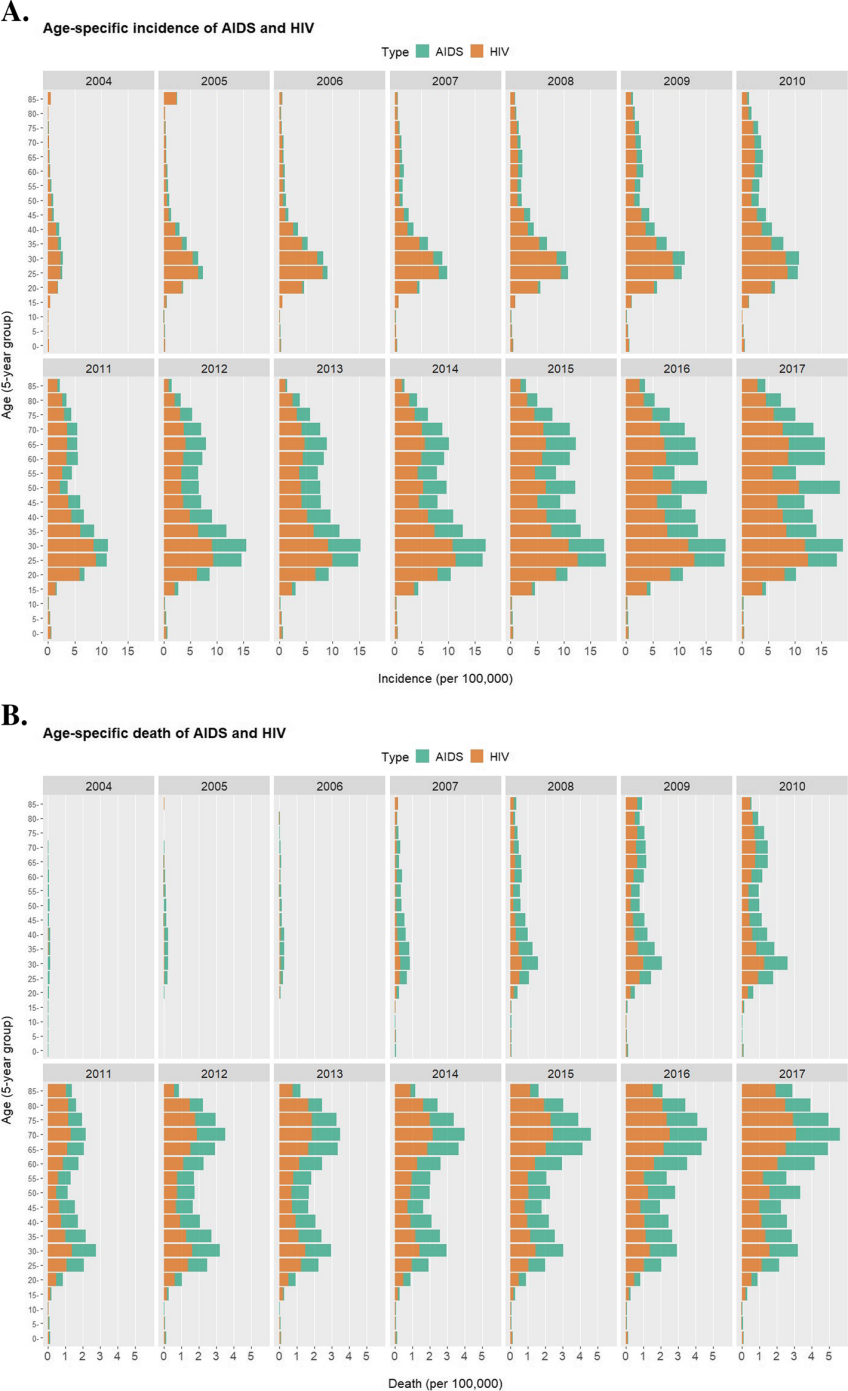
Age-specific incidence (**a**) and death (**b**) (per 100,000) of AIDS/HIV in China from 2004 to 2017

HIV could be transmitted from a patient/carrier to a healthy person by various means, so it is essential to differentiate the high-risk subgroups from the general population, especially regarding the age. Indeed, people belonging to different age groups demonstrate significantly disparate incidence (Fig. 4a) and death (Fig. 4b) of AIDS/HIV. The 0–14 years-old have low incidence because they are not yet prone to infection due to sexual activities or drug use. When teenagers mature into adolescence and adulthood, incidence of AIDS/HIV start to spike, and the 25–34 years-old are with the highest incidence. During the period of 2004–2011, the 25–39 years-old are with the highest death, but starting from 2012, the 65–79 years-old become the second peak in death and are currently the group with the highest death.

### ARIMA analysis

**Fig. 5:**
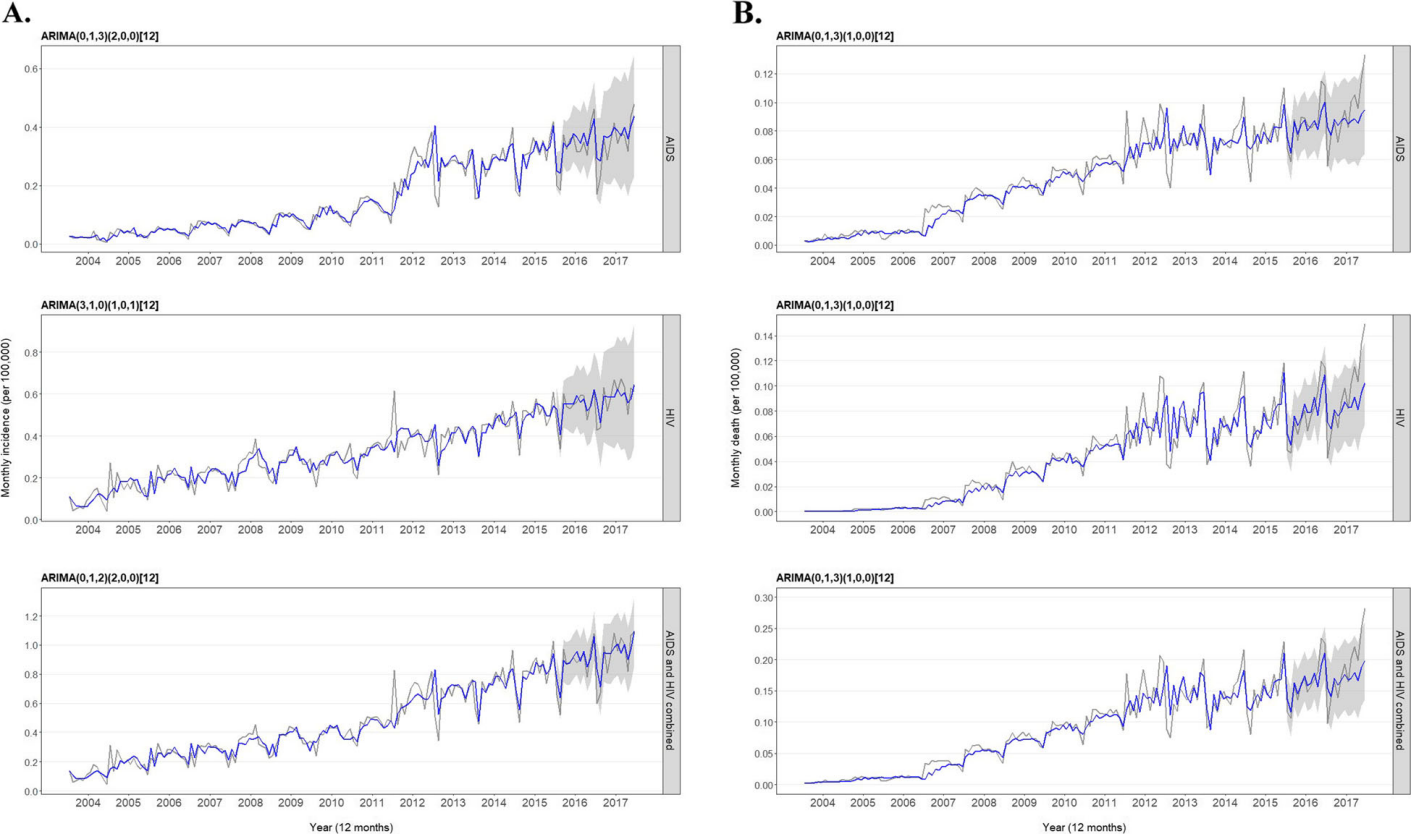
Monthly incidence (**a**) and death (**b**) (per 100,000) of AIDS/HIV and the two combined from 2004 to 2017 as reported (in gray) and fitted by the optimal ARIMA model (in blue). ARIMA models were fitted using data from 2004 to 2015, and parameters for the chosen ARIMA model were annotated in the title of individual time-series figures. ARIMA-based forecast of incidence or death in 2016 and 2017 displays a 95% prediction interval (gray ribbon). Note: the data are raw and fitted monthly incidence or death, and not annualized. For comparison to annual incidence or death, a multiplying factor of 12 should be used for monthly data. For example, monthly incidence of AIDS in January 2004 is 0.0272 per 100,000, and annual incidence of AIDS in 2004 is 0.2349 per 100,000

To extract and dissect the time trends of the incidence and death of AIDS/HIV, a statistical learning approach was applied to fit ARIMA models with data from 2004 to 2015 and use data in 2016 and 2017 for pseudo-forecast and model validation. For the fitting of respective models, a combinatorial spectrum of parameters were compared for ARIMA(p, d, q) and an optimal model was selected by the criteria of minimum AIC and BIC. For incidence, ARIMA (0, 1, 3) × (2, 0, 0), ARIMA (3, 1, 0) × (1, 0, 1), and ARIMA (0, 1, 2) × (2, 0, 0) were the optimal models for AIDS, HIV, and combined (Table 2 and Fig. 5a); for death, the identical ARIMA (0, 1, 3) × (1, 0, 0) was the optimal model for AIDS, HIV, and combined (Table 2 and Fig. 5b). Residuals in all of these fitted ARIMA models were pure random sequences (white noises) as confirmed by the Ljung-Box test (*P* > 0.05). ARIMA models fitted well with the historical 2004–2015 data and most importantly with the independent 2016–2017 data. One interesting observation worth attention was that the actual death data in the second half of 2017 were substantially higher than the corresponding ARIMA-fitted values, even surpassing the upper bound of the 95% prediction intervals for the last months of November and December in 2017. The ARIMA analysis demonstrates the practical applicability and translational values of the time series modeling in retrospectively extracting the underlying structure of AIDS/HIV trends, and as well in prospectively forecasting future incidence and death for the data-driven policy planning by public health authorities/agencies for the effective prevention and control of the infectious diseases.

**Table 2:** Goodness-of-fit test and selection of optimal ARIMA models

Time series	Optimal Model	Goodness-of-fit				Ljung-Box test	
		MASE	RMSE	AIC	BIC	χ^2^	*P* value
AIDS incidence	(0,1,3) × (2,0,0)_12_	0.55	0.03	− 553.19	− 535.42	0.00	0.99
HIV incidence	(3,1,0) × (1,0,1)_12_	0.69	0.05	− 436.07	− 418.29	0.09	0.77
AIDS and HIV incidence	(0,1,2) × (2,0,0)_12_	0.56	0.07	− 344.62	− 329.81	0.16	0.69
AIDS death	(0,1,3) × (1,0,0)_12_	0.62	0.01	− 926.73	− 911.92	0.05	0.83
HIV death	(0,1,3) × (1,0,0)_12_	0.61	0.01	− 901.39	− 886.58	0.02	0.89
AIDS and HIV death	(0,1,3) × (1,0,0)_12_	0.60	0.02	− 724.45	− 709.64	0.03	0.87

Note: *MASE* Mean absolute scaled error, *RMSE* Root mean square error, *AIC* Akaike’s information criterion, *BIC* Bayesian information criterion

## Discussion

AIDS/HIV is a serious global public health problem with both morbidity and mortality rising quickly across many nations. In this study, we focused on the latest data on incidence and death of AIDS/HIV in China from 2004 to 2017. Given that there could be a relatively long lapse between HIV infection and AIDS onset, it is important to analyze the epidemiological trends of annual/monthly AIDS patients and HIV carriers separately and in parallel. We observed matching rises in the incidence and death of AIDS and HIV, revealing the transition of historic HIV carriers into novel AIDS patients and the appearance of novel HIV-infected carriers whom would surely forecast a grim outlook of AIDS/HIV in future.

AIDS/HIV is not a seasonal infectious disease, but this study reveals an interesting pattern for the incidence and death of AIDS/HIV in China: a drop in January/February is consistently observed. This is likely due to the unique effects of annual Chinese New Year which generally falls on late January or early February, during which national and provincial CDCs are not fully functional and most hospitals/clinical labs run on limited capacity, both leading to the artificial drops in AIDS/HIV incidence or death records.

There is clear geographical disparity among all provincial regions. The exact reasons require further detailed investigations, but the clusters of hotbed regions identified in this study may share some underlying features including higher rate of intravenous drug use, poorer economic conditions, fewer public health and therapeutic resources, higher percentage of unprotected sexual activities (both homo- and hetero-) etc. The province of Henan is geographically distant from other hotbed regions, and the high incidence and death here is likely due to the cross-infection during illegal paid blood collection practices without adequate screening and sterilization in the early 1990s [27, 28].

There also exists age-specific disparity among different age groups. Mother-to-child transmission is responsible for more than 90% of childhood HIV cases [29]. In 2003, China launched a program integrating and standardizing the prevention of mother-to-child transmission (PMTCT) of HIV [30]. Effectiveness in such efforts was mirrored by our observation of the consistently low incidence of AIDS/HIV in the 0–4 years-old group. The upward trends in AIDS/HIV apply to the elderly as well: there was almost neglectable incidence of AIDS/HIV for people aged 60+ in 2004, but situation for the senior groups has deteriorated rapidly since then. With more AIDS onset and reduced immune response, the aged group suffers from disproportionally high deaths. Noticeably, the incidence and death may even be underestimated for the elderly because younger people account for the majority of HIV Voluntary Counseling & Testing (average age of participants is only 29.7 years-old) [31].

ARIMA models robustly estimate and forecast the incidence and death of AIDS/HIV, demonstrating the applicable potentials of time series modeling in retrospectively extracting the underlying epidemiology of infectious diseases and prospectively forecast future development trends and patterns. Both long-term annual trends and short-term monthly fluctuations could be appropriately fitted by the established ARIMA models for the data-driven prevention and control of infectious diseases. Additionally, model-predicted outlier peaks could in advance alarm potential outbreaks and prepare for essential intervention. It should be noted that the established ARIMA models were built on historic data, so the models should be continuously updated and optimized when the latest data get released and previously current data now become historic. This could be demonstrated by our interesting observation that the actual death data in the second half of 2017 were substantially higher than the corresponding ARIMA-forecasted values, even surpassing the upper bound of the 95% prediction intervals for the last months of November and December in 2017. This deviation reveals the faster increase in AIDS/HIV death during the latest time range, which is not factored into current ARIMA models (based on 2004–2015 data) but should be updated into future ARIMA models when data in later years of 2016 and so on are included for modeling.

A few imitations lie in this study. First, the most recent incidence and death data on AIDS/HIV in 2018 and 2019 were not yet released in the database by the completion of this manuscript so the established ARIMA models may not be the most accurate for forecasting future trends of AIDS/HIV in China for 2020 and beyond. Second, we analyzed in details on the geographical and age-specific distribution of AIDS/HIV, but other important factors including gender, social status, economic condition, and transmission mode were either not recorded in the National Infectious Disease Surveillance System or not released in the public health database. Analysis stratified on such factors are essential for the better understanding of AIDS/HIV epidemiology in China. Third, ARIMA models are autocorrelation approaches with limited ability to forecast outbreaks that happen in random frequencies. Additionally, just like all predictive models, uncertainty for forecasts grows rapidly with longer years into the future, so cautions should be taken using ARIMA models for long-term forecasting.

## Conclusion

Based on the spatial, age-wise, and temporal characteristics of AIDS/HIV epidemiology in China from 2004 to 2017, public health measures should be formulated to focus on the identified hotbed regions and high-risk age groups. ARIMA models fit well with the incidence and death of AIDS/HIV and the model-driven forecasts contribute to evidence-based policy decisions.

## Supplementary Information

**Additional file 1: Figure S1.** Annual total incidence and death counts of AIDS/HIV in China from 2004 to 2017. **Figure S2.** Monthly incidence (A) and death (B) counts (per 100,000) of AIDS/HIV in China from 2004 to 2017. **Figure S3.** Incidence (A) and death (B) (per 100,000) of AIDS/HIV in 31 provincial regions in odd years from 2005 to 2015 as gradient-scaled maps of China. Note: these maps were placed here to save space for Fig. 3, and thus the two figures should be interpreted together. Shaded provincial regions were comparable in Fig. 3 and Fig. S3 as the same color scale was applied in these two figures. **Figure S4.** Map of China annotated for provincial regions with high incidence or death of AIDS/HIV.

## Data Availability

- All data are accessible from the Data Center of China Public Health Science database (http://www.phsciencedata.cn/Share/en/) by registering a log-in account and selecting “typhoid and paratyphoid” for the type of infectious diseases.

## Abbreviations

HIV: Human immunodeficiency virus
AIDS: Acquired immunodeficiency syndrome
ARIMA: Autoregressive integrated moving average
AIC: Akaike’s information criterion
BIC: Bayesian information criterion
