# Supplementary material for: Epidemiological and time series analysis on the incidence and death of AIDS and HIV in China
Source: BMC Public Health. 2020 Dec 14;20:1906. doi: 10.1186/s12889-020-09977-8 (PMC7734828; doi:10.1186/s12889-020-09977-8)
Supplement: Supplementary file 1 — Additional file 1: Figure S1. Annual total incidence and death counts of AIDS/HIV in China from 2004 to 2017. Figure S2. Monthly incidence (A) and death (B) counts (per 100,000) of AIDS/HIV in China from 2004 to 2017. Figure S3. Incidence (A) and death (B) (per 100,000) of AIDS/HIV in 31 provincial regions in odd years from 2005 to 2015 as gradient-scaled maps of China. Note: these maps were placed here to save space for Fig. 3, and thus the two figures should be interpreted together. Shaded provincial regions were comparable in Fig. 3 and Fig. S3 as the same color scale was applied in these two figures. Figure S4. Map of China annotated for provincial regions with high incidence or death of AIDS/HIV. [file 12889_2020_9977_MOESM1_ESM.docx]

**Supplementary figures**

**Figure S1**


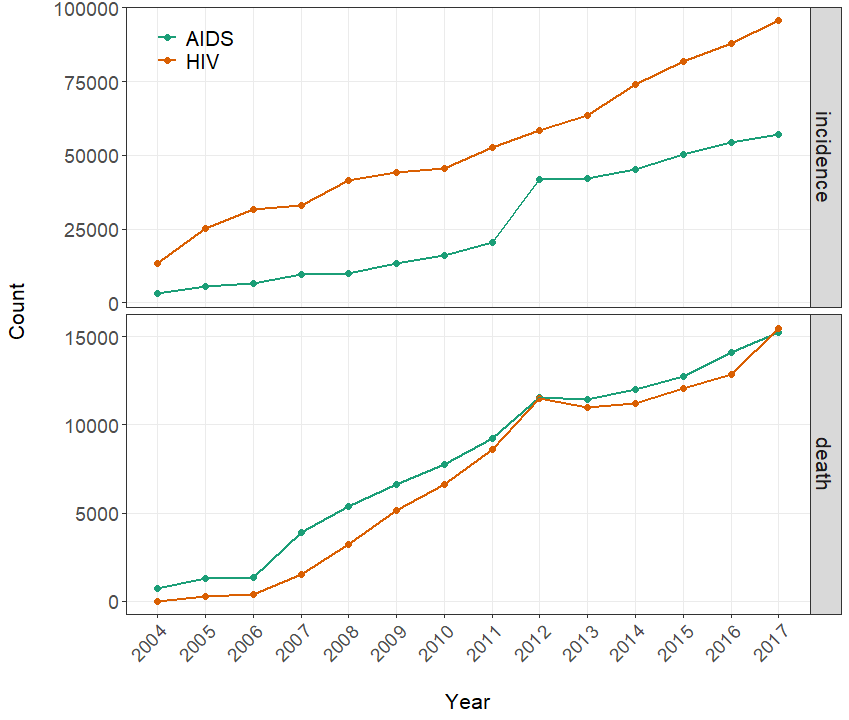


**Figure S2**

**A. B.**


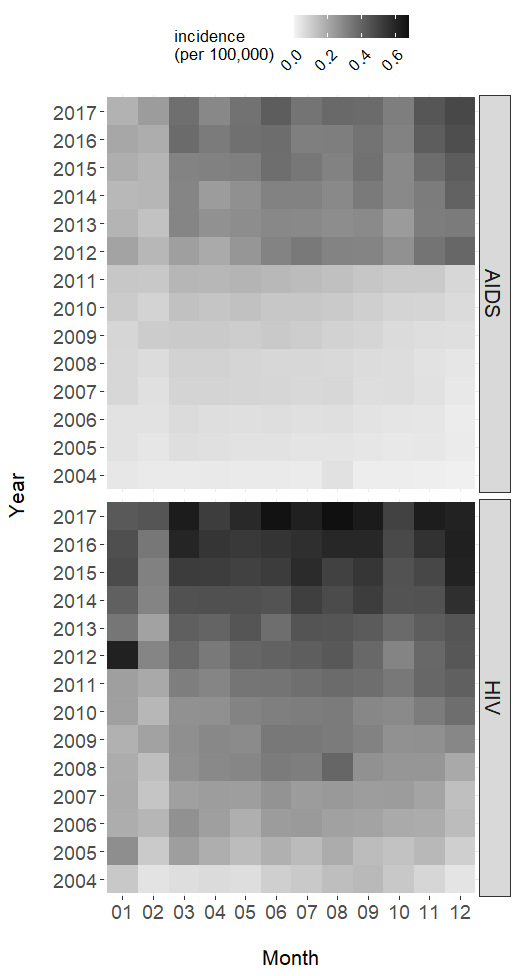

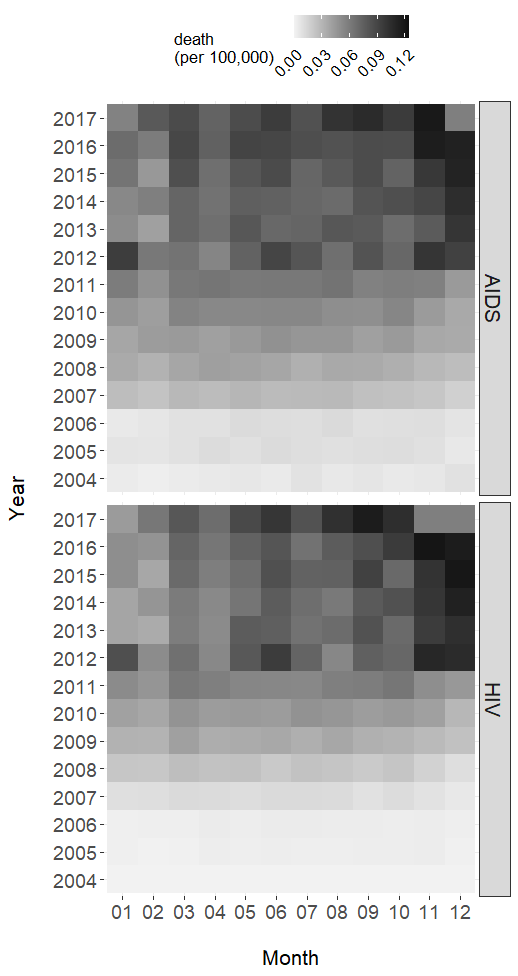


**Figure S3**

**A. B.**


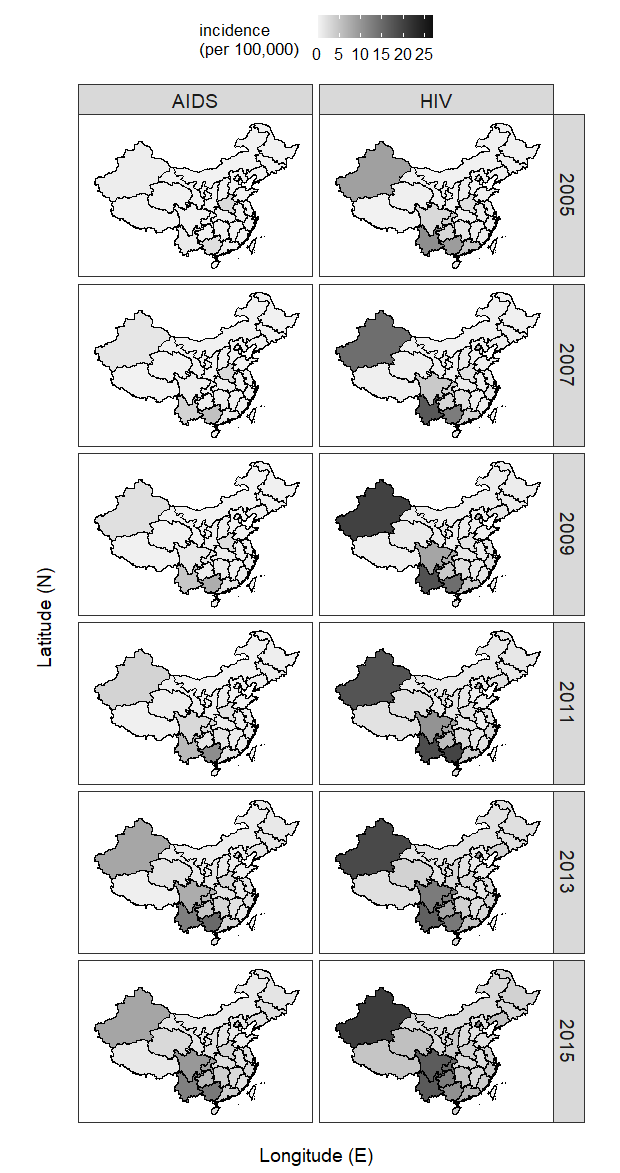

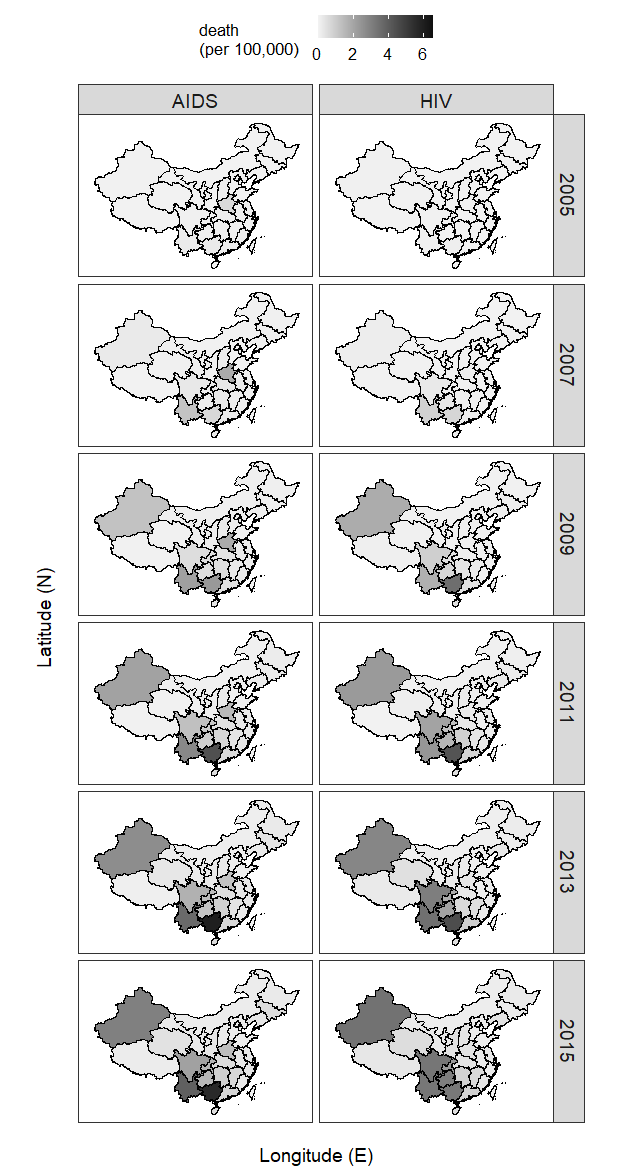


**Figure S4**


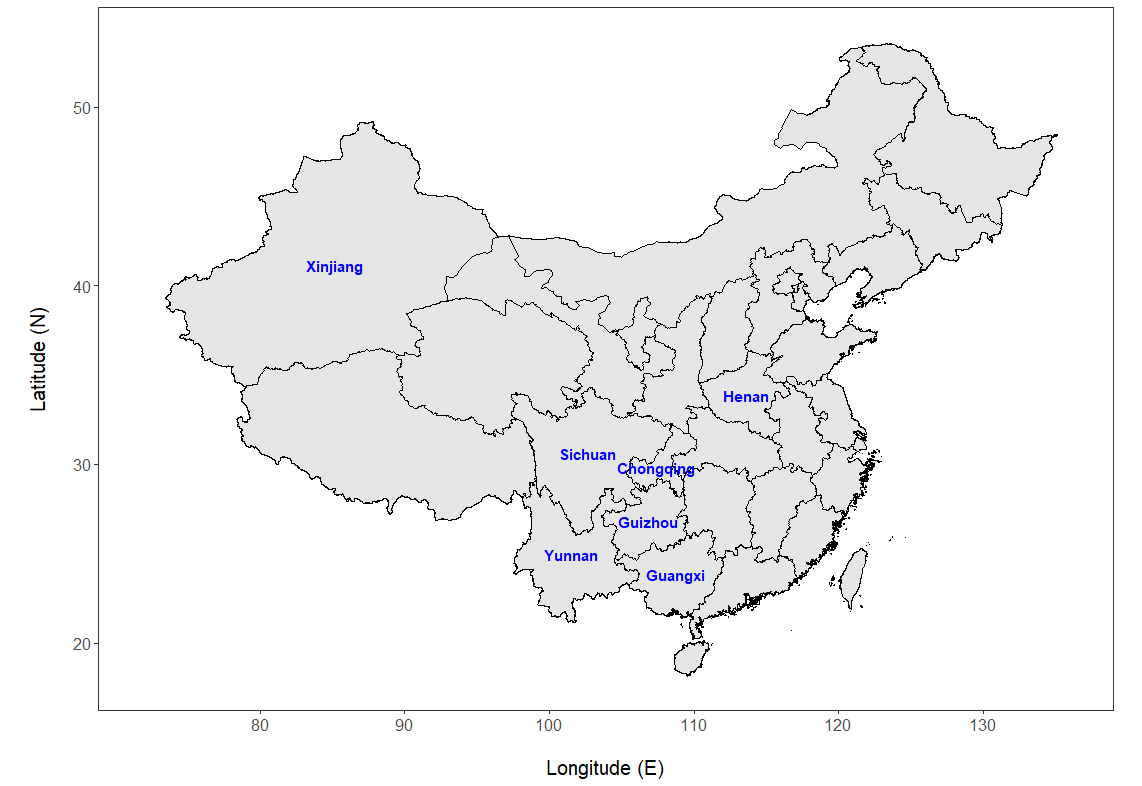


**Supplementary figure legends**

Figure S1. Annual total incidence and death counts of AIDS/HIV in China from 2004 to 2017.

Figure S2. Monthly incidence (A) and death (B) counts (per 100,000) of AIDS/HIV in China from 2004 to 2017.

Figure S3. Incidence (A) and death (B) (per 100,000) of AIDS/HIV in 31 provincial regions in odd years from 2005 to 2015 as gradient-scaled maps of China. Note: these maps were placed here to save space for Figure 3, and thus the two figures should be interpreted together. Shaded provincial regions were comparable in Fig. 3 and Fig. S3 as the same color scale was applied in these two figures.

Figure S4. Map of China annotated for provincial regions with high incidence or death of AIDS/HIV.
